# Draft Genome Sequence of an *Escherichia coli* O157:H43 Strain Isolated from Cattle

**DOI:** 10.1128/genomeA.00263-13

**Published:** 2013-05-30

**Authors:** Domonkos Sváb, Balázs Horváth, Attila Szűcs, Gergely Maróti, István Tóth

**Affiliations:** Institute for Veterinary Medical Research, Centre for Agricultural Research, Hungarian Academy of Sciences, Budapest, Hungarya; Biological Research Centre, Institute of Biochemistry, Hungarian Academy of Sciences, Szeged, Hungaryb

## Abstract

Here we report the draft genome sequence of an *Escherichia coli* O157:H43 strain, designated T22, with an atypical virulence gene profile and isolated from healthy cattle. T22 produces cytolethal distending toxin V (CDT-V) and belongs to phylogenetic group B1 and sequence type 155 (ST155).

## GENOME ANNOUNCEMENT

Enterohemorrhagic *Escherichia coli* (EHEC) strain O157:H7 has become a significant food-borne pathogen since its discovery (1). Additionally, strains of the serotype O157:NM (nonmotile) caused severe outbreaks in Europe (2). Typical EHEC O157 strains produce Shiga toxin (Stx) and carry the locus of enterocyte effacement (LEE) pathogenicity island encoding the adhesin intimin (3). Due to their clinical and epidemiological significance, the whole genomes of several outbreak strains have been fully sequenced (4, 5, 6). However, there are fewer data available on Stx-negative O157 strains and O157 strains that express flagellar antigens other than H7.

Recently, we identified atypical *E. coli* O157:H43 strains from healthy cattle that harbor neither *stx* nor *eae* virulence genes but that produce cytolethal distending toxin V (CDT-V), carry the genes encoding long polar fimbriae (*lpf2*), and belong to phylogenetic group B1 (7, 8).

The present study announces the first assembled draft genome of an *E. coli* O157:H43 strain. Chromosomal and plasmid DNA of an overnight culture of strain T22 of *E. coli* O157:H43 was isolated by GenElute bacterial genomic DNA kit (Sigma-Aldrich) and alkaline lysis, respectively. The chromosome and the plasmid DNA was sequenced and assembled using the combination of three next-generation sequencing (NGS) approaches (high-quality trimmed reads generated on the Life Technologies SOLiD V4 and Ion Torrent PGM as well as Roche’s 454 Titanium). Altogether, 6,994,992 SOLiD mate-paired reads of 50 plus 50 bp were combined with 233,333 Ion Torrent reads (mean read length, 161.1) and 73,764 reads from 454 Titanium (mean read length, 619). Trimming and assembly were performed manually using CLC Genomics Workbench 6.0 (9). The draft genome of T22 consists of a circular 4,959,535-bp chromosome and a plasmid with a length of 80,112 bp. The bacterial chromosome was assembled into 64 contigs containing 5,587 predicted genes (open reading frames [ORFs]) coding for 5,511 proteins, 17 rRNAs, and 59 tRNAs, while the plasmid was covered by 8 contigs harboring 90 genes (89 coding sequences [CDS] and 1 tRNA). Annotation was added by NCBI Prokaryotic Genomes Automatic Annotation Pipeline, which utilizes GeneMark, Glimmer, and tRNAscan-SE searches. The G+C content of the genome is 50.8%. None of the key virulence genes (10) of enterohemorrhagic (*stx, eae*), enteropathogenic (*eae, bfp*), enterotoxigenic (*lt, st*), enteroinvasive (*ihaH*), or enteroaggregative (*aggR*) *E. coli* strains were observed in the T22 genome. All the known integration sites of Stx phages (5, 10, 11) and LEE pathogenicity islands (12) were intact in the T22 chromosome. Multilocus sequence typing (MLST) analysis (13) based on the nucleotide sequences of seven housekeeping genes revealed that T22 belongs to the group sequence type 155 (ST155). These data could provide useful information for better understanding the evolution of *E. coli* O157.

### Nucleotide sequence accession numbers.

This Whole-Genome Shotgun project has been deposited at DDBJ/EMBL/GenBank under the accession no. AHZD00000000. The version described in this paper is the second version, accession no. AHZD02000000.

## References

[1] Riley LW Remis RS Helgerson SD McGee HB Wells JG Davis BR Hebert RJ Olcott ES Johnson LM Hargrett NT Blake PA Cohen ML 1983 Hemorrhagic colitis associated with a rare *Escherichia coli* serotype. N. Engl. J. Med. 308:681–685 6338386 10.1056/NEJM198303243081203

[2] Karch H Böhm H Schmidt H Gunzer F Aleksic S Heesemann J 1993 Clonal structure and pathogenicity of Shiga-like toxin-producing, sorbitol-fermenting *Escherichia coli* O157:H−. J. Clin. Microbiol. 31:1200–1205 8501218 10.1128/jcm.31.5.1200-1205.1993 PMC262903

[3] Kaper JB Nataro JP Mobley HL 2004 Pathogenic *Escherichia coli*. Nat. Rev. Microbiol. 2:123–140 15040260 10.1038/nrmicro818

[4] Hayashi T Makino K Ohnishi M Kurokawa K Ishii K Yokoyama K Han CG Ohtsubo E Nakayama K Murata T Tanaka M Tobe T Iida T Takami H Honda T Sasakawa C Ogasawara N Yasunaga T Kuhara S Shiba T Hattori M Shinagawa H 2001 Complete genome sequence of enterohemorrhagic *Escherichia coli* O157:H7 and genomic comparison with a laboratory strain K-12. DNA Res. 8:11–22 11258796 10.1093/dnares/8.1.11

[5] Perna NT Plunkett G III Burland V Mau B Glasner JD Rose DJ Mayhew GF Evans PS Gregor J Kirkpatrick HA Pósfai G Hackett J Klink S Boutin A Shao Y Miller L Grotbeck EJ Wayne Davis N Lim A Dimalanta ET Potamousis KD Apodaca J Anantharaman TS Lin J Yen G Schwartz DC Welch RA Blattner FR 2001 Genome sequence of enterohaemorrhagic *Escherichia coli* O157 H7. Nature 409:529–533 11206551 10.1038/35054089

[6] Eppinger M Daugherty S Agrawal S Galens K Sengamalay N Sadzewicz L Tallon L Cebula TA Mammel MK Feng P Soderlund R Tarr PI DebRoy C Dudley EG Fraser CM Ravel J 2013 Whole-genome draft sequences of 26 enterohemorrhagic *Escherichia coli* O157:H7 strains. Genome Announc. 1(2):e00134-12. doi:10.1128/genomeA.00134-12 10.1128/genomeA.00134-12 PMC3593324 23516226

[7] Tóth I Schmidt H Kardos G Lancz Z Creuzburg K Damjanova I Pászti J Beutin L Nagy B 2009 Virulence genes and molecular typing of different groups of *Escherichia coli* O157 strains in cattle. Appl. Environ. Microbiol. 75:6282–6291 19684174 10.1128/AEM.00873-09 PMC2753057

[8] Sváb D Tóth I 2012 Allelic types of long polar fimbriae in bovine and human *Escherichia coli* O157 strains. Acta Vet. Hung. 60:1–15 22366128 10.1556/AVet.2012.001

[9] Csuros M Juhos S Berces A 2010 Fast mapping and precise alignment of AB SOLiD color reads to reference DNA, p 176–188 *In* Moulton V Singh M, Proceedings of the 10th International Conference on Algorithms in Bioinformatics. Springer-Verlag, Berlin, Germany

[10] Ohnishi M Terajima J Kurokawa K Nakayama K Murata T Tamura K Ogura Y Watanabe H Hayashi T 2002 Genomic diversity of enterohemorrhagic *Escherichia coli* O157 revealed by whole-genome PCR scanning. Proc. Natl. Acad. Sci. U. S. A. 99:17043–17048 12481030 10.1073/pnas.262441699 PMC139266

[11] Recktenwald J Schmidt H 2002 The nucleotide sequence of Shiga toxin (Stx) 2e-encoding phage phiP27 is not related to other Stx phage genomes, but the modular genetic structure is conserved. Infect. Immun. 70:1896–1908 11895953 10.1128/IAI.70.4.1896-1908.2002 PMC127862

[12] Bertin Y Boukhors K Livrelli V Martin C 2004 Localization of the insertion site and pathotype determination of the locus of enterocyte effacement of Shiga toxin-producing *Escherichia coli* strains. Appl. Environ. Microbiol. 70:61–68 14711626 10.1128/AEM.70.1.61-68.2004 PMC321293

[13] Wirth T Falush D Lan R Colles F Mensa P Wieler LH Karch H Reeves PR Maiden MC Ochman H Achtman M 2006 Sex and virulence in *Escherichia coli*: an evolutionary perspective. Mol. Microbiol. 60:1136–1151 16689791 10.1111/j.1365-2958.2006.05172.x PMC1557465

